# Relationship Between Insoluble Dietary Fiber Intake and Non-Restorative Sleep in Japanese Adults: A Cross-Sectional Analysis of the NHNS Japan, 2014 and 2018

**DOI:** 10.3390/nu17233749

**Published:** 2025-11-28

**Authors:** Momo Fushimi, Aoi Kawamura, Tomohiro Utsumi, Kentaro Nagao, Kentaro Matsui, Ayano Kimura, Sayaka Aritake-Okada, Takuya Yoshiike, Kenichi Kuriyama

**Affiliations:** 1 Department of Sleep–Wake Disorders, National Institute of Mental Health, National Center of Neurology and Psychiatry, 4-1-1 Ogawa-Higashi, Kodaira, Tokyo 187-8553, Japanyoshiike@ncnp.go.jp (T.Y.); 2 Graduate School of Health and Social Services/School of Health Sciences, Saitama Prefectural University, 820 Sannomiya, Koshigaya, Saitama 343-8540, Japan

**Keywords:** insoluble dietary fiber, non-restorative sleep, sleep quality, dietary intake

## Abstract

Background/Objectives: Non-restorative sleep (NRS)—a subjective feeling of unrefreshing/insufficient rest despite adequate sleep duration—an important sleep-quality indicator is associated with depression and mortality. We examined whether insoluble dietary fiber intake is associated with NRS in Japanese adults, after adjusting for socioeconomic, lifestyle, and dietary factors. Methods: Using cross-sectional data from 5034 adult (≥20 years) respondents of the National Health and Nutrition Surveys (2014 and 2018), Japan, self-assessed NRS (coded as 1, absence as 0) was analyzed with insoluble fiber intake (g/1000 kcal) modeled as a continuous variable; logistic regression analyses with progressive adjustments included Model 1, adjusted for sex, age, and body mass index; Model 2, comprising socioeconomic and lifestyle factors; Model 3, adjusted for sleep duration; and Model 4, which included dietary energy, protein, fat, vitamin D, and magnesium. Results: Among 19.3% of participants with NRS, the median (interquartile range) insoluble dietary fiber intake was 5.45 (4.10–6.97) g/1000 kcal, with higher NRS prevalence among younger adults (<60 years), non-drinkers (no habitual alcohol consumption), and individuals with short sleep (<6 h). Higher insoluble dietary fiber intake was consistently associated with a lower NRS likelihood, before and after adjustment for potential confounders. In the fully adjusted model, younger age (<60 years), no habitual alcohol consumption, and short sleep (<6 h) were independently associated with greater odds of NRS. Conclusions: Higher insoluble dietary fiber density correlated with lower odds of NRS, indicating a significant association, rather than causation, that warrants longitudinal clarification of the temporal relationship between dietary fiber intake and perceived sleep restorativeness.

## 1. Introduction

Sleep is a fundamental biological process that is indispensable for restoring physical and mental functions and maintaining overall health. Insufficient or poor-quality sleep has been linked to increased risks of obesity, hypertension, diabetes, and depression, which highlights the importance of both adequate sleep duration and quality. According to the Diagnostic and Statistical Manual of Mental Disorders, Fifth Edition (DSM-5), non-restorative sleep (NRS) is not classified as an independent sleep–wake disorder, but rather represents a subjective symptom that reflects poor sleep quality, which is frequently observed as part of insomnia-associated disorders. It refers to an unrefreshing feeling after apparently sufficient sleep. Population-based studies indicate that NRS is common, with a prevalence approaching 10–30% in adults and is generally higher in women than in men [1]. Moreover, estimates vary by the assessment method—ranging from 2.4 to 16.1% in multi-national telephonic surveys to 19–31% in men and 26–42% in women when assessed via yes/no questionnaires [2]. NRS has been associated with higher risks of mortality and depression [3].

These previous findings highlight the need to clarify lifestyle factors that might affect NRS, with diet being one of the most modifiable and biologically plausible candidates. Although dietary patterns have been linked to general sleep regulation, the extent to which they specifically influence NRS remains unclear. Furthermore, the factors that contribute to NRS remain incompletely elucidated, and limited evidence is available regarding the role of specific dietary components in relation to NRS. Identifying modifiable lifestyle factors could therefore provide important insights for improving sleep quality at the population level.

Among dietary factors, several studies have suggested that daily intake of dietary fiber may not only contribute to the prevention of chronic lifestyle diseases [4,5] but also improve nighttime sleep quality [6].

Nonetheless, the majority of previous studies have focused on total fiber intake, and only a few have separately examined soluble and insoluble fiber intake in relation to sleep outcomes. Notably, a recent epidemiologic study in patients undergoing hemodialysis reported that higher insoluble fiber intake was independently associated with a lower prevalence of poor subjective sleep quality, which suggests a potential role of insoluble fiber intake in sleep regulation [7]. Considering the limited evidence specific to insoluble fiber, further investigation is warranted to clarify its relevance to NRS.

In addition to this limited epidemiologic evidence, certain insoluble fibers, such as cellulose, which is abundant in foods such as legumes, mushrooms, and vegetables, may promote melatonin secretion, whereas inadequate intake of insoluble fibers may be associated with sleep disturbances, possibly due to melatonin dysfunction [8,9]. These findings raise the possibility that the intake of insoluble dietary fiber plays a beneficial role in sleep quality. Moreover, previous research has indicated that sleep quality and dietary behaviors are influenced by socioeconomic status and lifestyle factors [10,11,12,13,14,15,16]. These factors may confound the association between dietary fiber intake and sleep quality, making it important to account for them in the analysis.

In this study, we examined the relationship between the intake of insoluble dietary fiber and NRS, after adjusting for potential confounders such as socioeconomic and lifestyle factors. We hypothesized that higher intake of insoluble dietary fiber is associated with a lower prevalence of NRS.

## 2. Materials and Methods

### 2.1. Study Design, Setting, and Duration

This cross-sectional study utilized data from the NHNS of Japan conducted in 2014 and 2018. The NHNS is an annual national health and nutrition survey carried out by the Ministry of Health, Labor, and Welfare of Japan under the Health Promotion Act. It includes physical examinations, one-day semi-weighed household dietary records, and lifestyle questionnaires. In this method, a household member records all foods and beverages consumed, with portion sizes either weighed or estimated. Trained dietitians then review the records, and nutrient intakes are calculated using the Standard Tables of Food Composition in Japan [17]. After reviewing all available NHNS datasets, we selected the 2014 and 2018 surveys because they were extended survey years that included a comprehensive set of variables essential for this study, given their established associations with both sleep quality and dietary patterns. These variables comprised NRS, insoluble dietary fiber intake, household income, occupation, household size, smoking status, alcohol consumption, body mass index (BMI), and sleep duration, as well as key dietary covariates, such as total energy intake, protein, fat, vitamin D, and magnesium [10,11]. Combining these two years also helped to minimize the potential influence of year-specific factors on the results.

### 2.2. Study Dataset

NRS has been surveyed in the National Health and Nutrition Survey (NHNS) [18]—an annual, large-scale, nationwide survey conducted by the Japan’s Ministry of Health, Labor and Welfare under the National Health Promotion Act. The NHNS was established in 2003, following the enactment of the Health Promotion Act in 2001 and replaced the former National Nutrition Survey, Japan (NNS-J). Since its inception in 2003, the NHNS has surveyed approximately 6000 households each year, and these households are selected through stratified random sampling. The NHNS thus provides essential data for promoting public health in Japan and covers various aspects of dietary patterns and health status, including sleep health, that is surveyed using self-perceived NRS [19,20].

**Figure 1:**
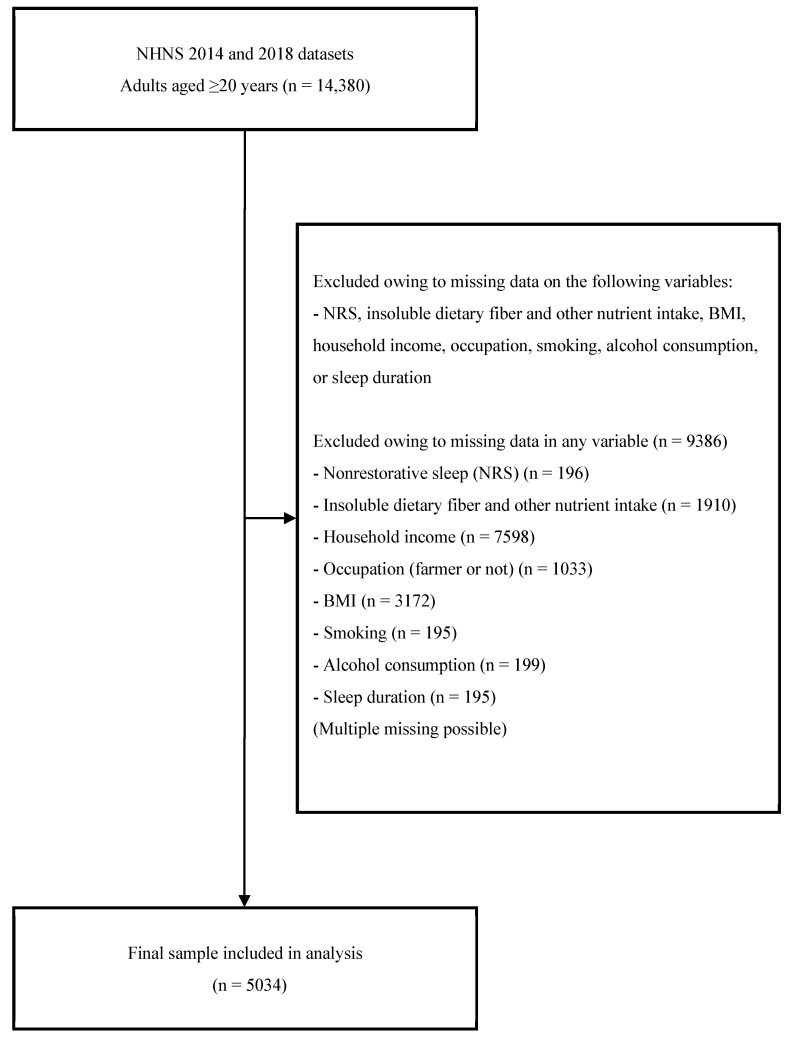
Flow diagram of participant selection from the NHNS 2014 and 2018 datasets [21,22]. Participants aged ≥20 years from the 2014 and the 2018 National Health and Nutrition Surveys (NHNS) were included in the study (*n* = 14,380). Individuals with missing values in any of the following variables were excluded: non-restorative sleep (NRS), insoluble dietary fiber and other nutrient intakes, body mass index (BMI), household income, occupation, smoking, alcohol consumption, or sleep duration. The final sample comprised 5034 participants.

In total, 14,380 adults aged ≥20 years were eligible (7738 from the 2014 survey and 6642 from the 2018 survey). The NHNS targets individuals aged ≥20 years under Japan’s national health policy for preventing lifestyle-related diseases. We excluded individuals with missing data for any study variable (Figure 1), leaving a complete-case dataset for all variables under investigation.

### 2.3. Survey Items

#### 2.3.1. Outcomes

NRS was assessed using a self-rated four-point scale based on the question: “Have you been getting enough restorative sleep in the past month?” The four response options were: (1) very much, (2) yes, (3) not much, and (4) not at all. In accordance with previous studies [23,24], responses were dichotomized, and the participants were stratified into two groups: NRS-present (scores 3–4; “not much” or “not at all”) and NRS-absent (scores 1–2; “very much” or “yes”). For subsequent analyses, the presence of NRS was coded as 1 and its absence as 0.

#### 2.3.2. Exposure

In the NHNS, dietary variables were derived from the 1-day semi-weighed household dietary record based on the proportional distribution method (proportional division method) that has been used since 1995. The main recordkeepers, who usually prepared meals in the household, recorded all foods and beverages consumed by each household member, including the amounts prepared, waste, and leftovers. When multiple family members shared a dish, the recordkeepers recorded the approximate proportion consumed by each individual (e.g., as percentages or ratios). For occasions where the respondents ate outside, the portion sizes and leftovers were self-reported by the participants [25]. All food items were coded according to the Standard Tables of Food Composition in Japan and linked to the NHNS Food Number Table, which standardizes food coding and nutrient calculation. Nutrient intakes, including dietary fiber, were calculated based on these tables, and insoluble dietary fiber intake was defined as the total amount of insoluble fiber consumed from all foods on the survey day. The classification of foods as sources of insoluble dietary fiber (e.g., cereals, pulses, vegetables, mushrooms, and seaweeds) was based on the NHNS food group coding system. The fiber values for each food were obtained from analytical data included in the Standard Tables of Food Composition in Japan.

Dietary intake of insoluble fiber was adjusted for total energy intake using the nutrition density method and expressed as grams per 1000 kilocalories [26,27]. Total energy intake is presented in kilocalories per day. Protein and fat intakes are expressed as percentages of the total energy intake (% energy). Similarly, vitamin D and magnesium intakes were also adjusted for energy using the density method and expressed as µg/1000 kcal and mg/1000 kcal, respectively. This approach allows for the comparison of nutrient intake independent of total energy consumption and is commonly used in nutritional epidemiology to control for confounding by energy intake.

#### 2.3.3. Study Covariates

Demographic factors included age, sex, and body mass index (BMI). Age was recorded as a continuous variable and categorized into two groups for analysis: <60 and ≥60 years. Sex was recorded as male or female, and BMI was obtained as a continuous variable.

Several potential confounders, such as socioeconomic status, lifestyle, and sleep duration, were considered, as these factors are known to influence both dietary patterns and sleep quality. For example, individuals with higher socioeconomic status tend to consume more dietary fiber [12] and report better sleep quality [16]. Conversely, lifestyle factors such as smoking and alcohol consumption are associated with both lower dietary fiber intake [13,14] and poorer sleep quality [28,29]. In addition, sleep duration plays a critical role in determining sleep quality and may also influence dietary behaviors, including dietary fiber intake [15,30]. These confounding factors make it difficult to isolate the direct influence of dietary fiber intake on sleep quality. To address this issue, we adjusted for relevant covariates across four models as detailed below.

Socioeconomic status was assessed using three indicators: household size, household income, and occupation. Household size was dichotomized into one-person and two-or-more-person households. Household income was classified into three categories: low (<2 million yen), middle (2 to <6 million yen), and high (≥6 million yen) [26]. Occupation was categorized as agricultural worker or non-agricultural worker, as agricultural lifestyles may affect dietary fiber intakes [26].

Lifestyle factors included habitual smoking and alcohol consumption. Habitual smoking was defined as individuals who responded that they “smoke every day” or “smoke occasionally.” Habitual alcohol consumption was defined based on the criteria of the NHNS as drinking alcohol three or more days per week and consuming at least one *go* (180 mL) of sake per day, which equated to approximately 20 g of pure alcohol per day.

Daily sleep duration was assessed using the question, “How many hours did you sleep on average per day during the past month?” Participants were asked to choose from the following six categories: <5 h, 5 to 6 h, 6 to 7 h, 7 to 8 h, 8 to 9 h and >9 h. These responses were reclassified into three categories: <6 h, 6 to 8 h, and >8 h.

### 2.4. Statistical Analysis

Logistic regression analysis was used to examine the relationship between NRS (outcome) and insoluble dietary fiber intake.

In addition to the unadjusted model, we used four multivariable-adjusted models. Model 1 adjusted for sex, age, and BMI. Model 2 further included socioeconomic status (household size, household income, and occupation), and lifestyle factors (habitual smoking and alcohol consumption), given their established association with dietary fiber intake or NRS. Model 3 further adjusted for sleep duration, which is strongly associated with NRS and may also relate to dietary fiber intake. Model 4 additionally adjusted for dietary factors, including total energy intake (kcal/day), protein and fat (% energy), and micronutrients, such as vitamin D and magnesium (per 1000 kcal), all of which may influence both sleep quality and dietary behavior.

We evaluated multicollinearity among the independent variables using the variance inflation factor (VIF), with all VIF values of <3.0. All statistical analyses were conducted using IBM SPSS Statistics for Windows, version 23.0 (IBM Corp., Armonk, NY, USA). A *p*-value of <0.05 was considered statistically significant.

### 2.5. Sensitivity Analysis

We conducted several analyses to exclude other possible explanations for the association between NRS and insoluble dietary fiber intake. First, we conducted a sensitivity analysis incorporating the city size classification (five categories: government-designated cities, other large cities, medium-sized cities, small cities, and towns or villages) to account for potential bias arising from the stratified sampling design. Second, to account for potential bias arising from missing data, we compared the observed characteristics between included versus excluded participants. Furthermore, we created 20 datasets and imputed missing values via the multiple imputation method using chained equations, and we evaluated the association between NRS and insoluble dietary fiber intake under the assumption of missing at random. Third, we conducted ordinal logistic regression analysis treating NRS as a four-category ordinal variable to account for potential bias arising from reducing a polytomous variable to a binary outcome. Fourth, we conducted a sensitivity analysis using the residual method instead of the density approach. Fifth, we treated age as a continuous variable, rather than as a categorical variable, and included survey year as a covariate to account for the period effects spanning 2014 to 2018. Sixth, to account for potential bias arising from the dichotomization of occupation variables into agricultural and non-agricultural workers, we conducted a sensitivity analysis using employment status. Seventh, we conducted sensitivity analysis categorizing insoluble dietary fiber intake into quartiles to examine the dose–response association between insoluble dietary fiber intake and NRS.

## 3. Results

### 3.1. Sociodemographic and Lifestyle Characteristics of Participants

A total of 5034 individuals were included in the analysis. Participant characteristics stratified by NRS status, including demographic, socioeconomic, lifestyle, and sleep-related variables, are presented in Table 1.

### 3.2. Association Between Insoluble Dietary Fiber Intake and NRS

In the unadjusted model, a higher intake of insoluble dietary fiber was significantly associated with decreased odds of NRS (odds ratio [OR] = 0.88, 95% confidence interval [CI]: 0.85–0.91, *p* < 0.001). This association remained significant after adjusting for sex, age, and BMI in Model 1 (adjusted OR = 0.95, 95% CI: 0.91–0.98, *p* = 0.003), and persisted after further adjustment for socioeconomic and lifestyle factors in Model 2 (adjusted OR = 0.94, 95% CI: 0.90–0.97, *p* = 0.001). When sleep duration was added in Model 3, the association continued to hold (adjusted OR = 0.94, 95% CI: 0.91–0.98, *p* = 0.003). In the fully adjusted model (Model 4), which additionally accounted for dietary intake of energy, protein, fat, vitamin D, and magnesium, higher insoluble dietary fiber intake remained significantly associated with lower odds of NRS (adjusted OR = 0.95, 95% CI: 0.91–0.99, *p* = 0.024) (Table 2).

In the fully adjusted model, a higher insoluble dietary fiber intake was associated with lower odds of NRS, whereas older age (≥60 years), habitual alcohol consumption (≥180 mL of sake on ≥3 days per week), and short sleep duration (<6 h) were independently associated with increased odds of NRS.

### 3.3. Sensitivity Analyses Results

Across the sensitivity analyses, a higher intake of insoluble dietary fiber was consistently associated with decreased odds of NRS: that is, using the city-size classification (fully adjusted OR = 0.95, 95% CI: 0.91–0.99; Table S1) as a covariate, using the data supplemented via multiple imputation for missing values (*n* = 14,380, fully adjusted OR = 0.97, 95% CI: 0.94–1.00; Table S2), using the NRS as a four-category ordinal variable (Model 3 OR for NRS vs. restorative sleep = 0.87, 95% CI: 0.76–0.98; Table S3), using the residual method instead of the density approach (fully adjusted OR = 0.97, 95% CI: 0.95–1.00; Table S4). Moreover, the association persisted when we treated age as a continuous, rather than categorical, variable (fully adjusted OR = 0.95, 95% CI: 0.91–0.99; Table S5) and when using the employment status instead of a dichotomized variable (agricultural vs. non-agricultural fully adjusted OR = 0.95, 95% CI: 0.91–1.00; Table S6). Furthermore, the association persisted when categorizing insoluble dietary fiber intake into quartiles (Model 3 OR for highest vs. lowest quartile = 0.73, 95% CI: 0.58–0.93; Table S7).

## 4. Discussion

The current study found that a higher intake of insoluble dietary fiber was significantly associated with lower odds of experiencing NRS among Japanese adults, independent of other factors such as age, sex, BMI, and lifestyle habits. This finding suggests that inadequate consumption of insoluble fiber may directly contribute to subjective perceptions of poor sleep quality.

Importantly, the association between lower insoluble fiber intake and greater odds of NRS remained statistically significant, even after adjusting for dietary nutrients that were previously reported to influence sleep quality, including energy, protein, fat, vitamin D, and magnesium (Model 4). Therefore, the observed relationship was not potentially confounded by other nutritional factors, and insoluble fiber potentially has an independent role in promoting restorative sleep. Higher dietary fiber intake was consistently associated with better self-rated physical and mental health across diverse populations, including healthy young adults and individuals with depressive disorders [31,32,33,34]. Thus, dietary fiber potentially influences subjective well-being-including sleep-related perceptions. This association could be intermediated through multiple pathways, such as modulation of gut microbiota, improvements in intestinal barrier function, and reduced systemic inflammation, all of which mediate the regulation of perceived health and sleep quality [35,36,37]. As the NRS reflects a subjective aspect of sleep health, the observed association between lower insoluble fiber intake and greater odds of NRS in our study is consistent with broader evidence of the association between fiber consumption and improved perceived health.

In addition, younger age (<60 years) was independently associated with NRS in the fully adjusted model. This pattern may reflect the fact that working-age adults frequently experience greater psychosocial stress, longer working hours, and stronger work–family conflict [38], which can impair perceived sleep restorativeness, despite relatively preserved objective sleep architecture. Moreover, younger adults may have more irregular sleep–wake schedules, greater nighttime exposure to electronic devices, and a higher prevalence of mood and anxiety symptoms—all of which worsen subjective sleep quality [39,40].

In this study, participants without habitual alcohol consumption had higher odds of NRS than those who met the definition of habitual drinking. This counterintuitive association should not be interpreted as evidence that alcohol improves sleep. Rather, it may reflect residual confounding or reverse causation; for example, individuals with existing sleep or health problems, or those taking medications, may avoid alcohol, whereas some people with sleep difficulties may use alcohol as a self-medication strategy that is not fully captured by our categorical definition of exposure. Experimental and clinical studies have consistently shown that alcohol disrupts sleep architecture by increasing sleep fragmentation, suppressing rapid eye movement sleep, and impairing sleep continuity; therefore, sleep is likely to feel less restorative despite any short-term sedative effects of alcohol [41].

Furthermore, insufficient sleep duration itself is associated with increased fatigue; poorer subjective sleep quality; and impairments in cognitive, emotional, and metabolic functioning [3,42]. Both aging and alcohol use are associated with shorter or more fragmented sleep and may therefore contribute to NRS—both directly and indirectly—by influencing sleep quantity and quality.

Taken together with the observed association between dietary fiber intake and NRS scores, these findings suggest that both physiological and behavioral factors, including age, alcohol use, sleep duration, and nutrition, may play important roles in determining subjective sleep quality.

However, this study has certain limitations that should be acknowledged. First, due to its cross-sectional design, causal inferences regarding the relationships between dietary fiber intake and NRS could not be established. Second, sleep duration was assessed using a self-reported questionnaire rather than objective measures. Subjective reports of sleep duration may be less accurate than objective data obtained via actigraphy or polysomnography, which can provide more precise estimates of actual sleep time and time in bed [43]. Previous studies have shown that objective short sleep duration, as opposed to perceived sleep duration, is strongly associated with physiological dysregulation and adverse health outcomes [44]. Third, potential residual confounding factors cannot be ruled out, despite adjusting for various lifestyle and dietary covariates. For example, other unmeasured dietary components or mental health factors, such as stress and depressive symptoms, as well as sleep disorders (e.g., sleep apnea and restless legs syndrome) and chronic medical conditions (e.g., cardiovascular disease, diabetes, and chronic pain), all of which are known to influence sleep quality, were not included in the models. Fourth, dietary assessment relied on a single 1-day semi-weighed household record, which is vulnerable to day-to-day variability and may not capture the usual intake, which potentially attenuates the observed associations. The lack of control for seasonality and day-of-week effects in NHNS data collection potentially introduces measurement errors. Finally, although the sample size was sufficiently large, the study population consisted exclusively of Japanese adults, which may limit the generalizability to other populations. In addition, because income had a relatively high proportion of missing values, excluding participants with incomplete data and other covariates reduced the analytical sample size. The comparison of included versus excluded participants showed similar distributions of age, BMI, and lifestyle factors; however, women were slightly overrepresented in the excluded group. Multiple imputation analysis (20 cycles using chained equations) produced results that were consistent with the main findings, which suggests robustness. However, residual selection bias remains possible, and generalizability may be limited to populations with complete survey data. This may have resulted in a partial reduction in national representativeness.

## 5. Conclusions

In light of these limitations, our findings nonetheless indicate that higher insoluble dietary fiber intake was independently associated with lower odds of experiencing NRS in this large population-based study, even after comprehensive adjustment for socioeconomic status, lifestyle, and sleep duration. These results suggest that insoluble fiber intake may be a relevant and potentially modifiable factor associated with perceived sleep restorativeness. Although the cross-sectional design precludes causal inference, the observed association, together with plausible mechanistic pathways linking insoluble fiber to melatonin dynamics and sleep regulation, supports the need for further longitudinal and interventional studies. Further research is needed to clarify the temporal relationship between insoluble fiber intake and NRS in conjunction with objective sleep variables, explore potential mediating biological pathways, and assess whether increasing insoluble fiber consumption could help improve individual sleep health in clinical and general populations.

## Figures and Tables

**Table 1:** Participant characteristics according to NRS status (*n* = 5034).

Variable	NRS (*n* = 974)	Restorative Sleep (*n* = 4060)	All (*n* = 5034)	Prevalence of NRS (%)
All	974	4060	5034	19.3
Sex, *n* (%)				
Female	251 (25.8)	1038 (25.6)	1289 (25.6)	19.5
Male	723 (74.2)	3022 (74.4)	3745 (74.4)	19.3
Age, *n* (%)				
<60 years	622 (63.9)	1385 (34.1)	2007 (39.9)	31.0
≥60 years	352 (36.1)	2675 (65.9)	3027 (60.1)	11.6
BMI, mean (SD)	23.44 (3.7)	23.36 (3.4)	23.40 (3.4)	
Household size *n* (%)				
1 person	218 (22.4)	956 (23.5)	1174 (23.3)	18.6
≥2 persons	756 (77.6)	3104 (76.5)	3860 (76.7)	19.6
Household income *n* (%)				
<2 million yen, low	181 (18.6)	958 (23.6)	1139 (22.6)	15.9
2–6 million yen, middle	471 (48.4)	2206 (54.3)	2677 (53.2)	17.6
≥6 million yen, high	322 (33.1)	896 (22.1)	1218 (24.2)	26.4
Occupation *n* (%)				
Non-agricultural workers	940 (96.5)	3847 (94.8)	4787 (95.1)	19.6
Agricultural workers	34 (3.5)	213 (5.2)	247 (4.9)	13.8
Habitual smoking *n* (%)				
No	715 (73.4)	3097 (76.3)	3812 (75.7)	18.8
Yes	259 (26.6)	963 (23.7)	1222 (24.3)	21.2
Habitual alcohol consumption *n* (%)				
No	619 (63.6)	2292 (56.5)	2911 (57.8)	21.3
Yes	355 (36.4)	1768 (43.5)	2123 (42.2)	16.7
Sleep duration *n* (%)				
<6 h	683 (70.1)	1093 (26.9)	1776 (35.3)	38.5
6–8 h	263 (27.0)	2506 (61.7)	2769 (55.0)	9.5
≥8 h	28 (2.9)	461 (11.4)	489 (9.7)	5.7
Insoluble dietary fiber intake (g/1000 kcal), mean (SD)	5.23 (2.2)	5.83 (2.3)	5.71 (2.3)	
Energy (kcal/day), mean (SD)	2028.09 (609.8)	2054.14 (562.8)	2049.10 (572.2)	
Protein (% energy), mean (SD)	14.54 (3.2)	14.76 (3.0)	14.72 (3.1)	
Fat (% energy), mean (SD)	27.04 (7.7)	25.94 (7.6)	26.16 (7.6)	
Vitamin D intake (µg/1000 kcal), mean (SD)	3.36 (4.3)	4.06 (4.5)	3.92 (4.5)	
Magnesium intake (mg/1000 kcal), mean (SD)	129.77 (44.4)	139.15 (44.7)	137.34 (44.7)	

Descriptive statistics of demographic, lifestyle, socioeconomic, and sleep-related variables among participants were classified as having NRS (*n* = 974) or restorative sleep (*n* = 4060) based on responses to the NRS question. Continuous variables are presented as mean (standard deviation) and categorical variables as number (percentage).

**Table 2:** Logistic regression analysis of factors associated with NRS.

Variable	Unadjusted OR (95% CI)	Model 1 AOR (95% CI)	Model 2 AOR (95% CI)	Model 3 AOR (95% CI)	Model 4 AOR (95% CI)
Insoluble dietary fiber (g/1000 kcal)	0.88 (0.85–0.91) ***	0.95 (0.91–0.98) **	0.94 (0.90–0.97) ***	0.94 (0.91–0.98) **	0.95 (0.91–0.99) *
Sex					
Male	Ref	Ref	Ref	Ref	Ref
Female	1.01 (0.86–1.19)	1.20 (1.01–1.43) *	–	–	–
Age					
<60 years	Ref	Ref	Ref	Ref	Ref
≥60 years	0.29 (0.25–0.34) ***	0.31 (0.27–0.36) ***	0.32 (0.27–0.38) ***	0.39 (0.32–0.46) ***	0.39 (0.33–0.47) ***
BMI	1.01 (0.99–1.03)	–	–	–	–
Household size					
1 person	Ref		Ref	Ref	Ref
≥2 persons	1.07 (0.90–1.26)		–	–	–
Household income					
<2 million yen	Ref		Ref	Ref	Ref
2–6 million yen	1.13 (0.94–1.36)		–	–	–
≥6 million yen	1.90 (1.55–2.33) ***		–	–	–
Occupation					
Non-agricultural	Ref		Ref	Ref	Ref
Agricultural	0.65 (0.45–0.94) *		–	–	–
Smoking status					
No	Ref		Ref	Ref	Ref
Yes	1.16 (0.99–1.37)		–	–	–
Alcohol consumption					
No	Ref		Ref	Ref	Ref
Yes	0.74 (0.64–0.86) ***		0.71 (0.61–0.83) ***	0.76 (0.64–0.89) ***	0.77 (0.65–0.91) **
Sleep duration					
<6 h	5.95 (5.08–6.98) ***			5.44 (4.62–6.41) ***	5.41 (4.59–6.37) ***
6–8 h	Ref			Ref	Ref
≥8 h	0.58 (0.39–0.87) **			–	–
Energy (kcal/day)	1.00 (1.00–1.00)				–
Protein (% energy)	0.98 (0.95–1.00) *				–
Fat (% energy)	1.02 (1.01–1.03) ***				–
Vitamin D intake (µg/1000 kcal)	0.96 (0.94–0.98) ***				–
Magnesium intake (mg/1000 kcal)	0.99 (0.99–1.00)***				–

Odds ratios (ORs) and 95% confidence intervals (CIs) for NRS (defined as the absence of sufficient restorative sleep) according to insoluble dietary fiber intake and covariates. Logistic regression models were used: the unadjusted model, Model 1 (adjusted for sex, age, and BMI), Model 2 (further adjusted for socioeconomic status and lifestyle factors), Model 3 (additionally adjusted for sleep duration), and Model 4 (further adjusted for dietary factors, including total energy intake, protein, fat, vitamin D, and magnesium intake). Statistical significance: * *p* < 0.05, ** *p* < 0.01, *** *p* < 0.001.

## Data Availability

The data used in this study are available from the Ministry of Health, Labor, and Welfare of Japan upon request. However, restrictions apply to the availability of these data, which were used under license for the purpose of this study and are not publicly available.

## Supplementary Materials

The following supporting information can be downloaded at: https://www.mdpi.com/article/10.3390/nu17233749/s1, Table S1: Logistic regression analysis of factors associated with non-restorative sleep, including adjustment for city size; Table S2: Logistic regression analysis of factors associated with non-restorative sleep using multiple imputation; Table S3: Multinomial logistic regression analyses of factors associated with non-restorative sleep; Table S4: Logistic regression analysis of factors associated with non-restorative sleep using energy-adjusted insoluble dietary fiber (residual method); Table S5: Logistic regression analysis of factors associated with non-restorative sleep, incorporating age as a continuous variable and survey year (2014–2018) as an additional covariate; Table S6: Logistic regression analysis of factors associated with non-restorative sleep using detailed occupation categories instead of dichotomized occupation status; Table S7: Logistic regression analysis of factors associated with non-restorative sleep using quartiles of insoluble dietary fiber intake.

## Abbreviations

The following abbreviations are used in this manuscript:

BMI	Body Mass Index
CI	Confidence Interval
IBM	International Business Machines Corporation
kcal	Kilocalorie(s)
mg	Milligram(s)
NHNS	National Health and Nutrition Survey
NNS-J	National Nutrition Survey, Japan
NRS	Non-Restorative Sleep
OR	Odds Ratio
REM	Rapid Eye Movement
SPSS	Statistical Package for the Social Sciences
VIF	Variance Inflation Factor
