# Supplementary material for: Relationship Between Insoluble Dietary Fiber Intake and Non-Restorative Sleep in Japanese Adults: A Cross-Sectional Analysis of the NHNS Japan, 2014 and 2018
Source: Nutrients. 2025 Nov 28;17(23):3749. doi: 10.3390/nu17233749 (PMC12694229; doi:10.3390/nu17233749)
Supplement: Supplementary file 1 [file nutrients-17-03749-s001.zip › nutrients-3969768-supplementary.pdf]

Table S1. Logistic regression analysis of factors associated with non-restorative sleep, including adjustment for city size.

Variable	Unadjusted OR (95%CI)	Model 1 AOR (95%CI)	Model 2 AOR (95%CI)	Model 3 AOR (95%CI)	Model 4 AOR (95%CI)
**Insoluble dietary fiber (g/1,000kcal)**	0.88 (0.85–0.91)***	0.95 (0.91–0.98)**	0.94 (0.90-0.97)***	0.94 (0.91–0.98)**	0.95 (0.91–0.99)*
**Sex**					
**Male**	Ref	Ref	Ref	Ref	Ref
**Female**	1.01 (0.86-1.19)	1.20 (1.01-1.43)*	–	–	–
**Age**					
**< 60 years**	Ref	Ref	Ref	Ref	Ref
**≥ 60 years**	0.29 (0.25-0.34)***	0.31 (0.27-0.36)***	0.32 (0.27-0.38)***	0.39 (0.32-0.46)***	0.39 (0.33-0.47)***
**BMI**	1.01 (0.99-1.03)	–	–	–	–
**Household size**					
**1 person**	Ref		Ref	Ref	Ref
**≥ 2 persons**	1.07 (0.90-1.26)		–	–	–
**Household income**					
**< 2 million yen**	Ref		Ref	Ref	Ref
**2–6 million yen**	1.13 (0.94-1.36)		–	–	–
**≥ 6 million yen**	1.90 (1.55-2.33)***		–	–	–
**City size**					
**government-designated cities**	Ref	Ref	Ref	Ref	Ref
**other large cities**	1.18 (0.97-1.42)	–	–	–	–
**medium-sized cities**	0.93 (0.76-1.14)	–	–	–	–
**small cities**	0.74 (0.54-1.03)	–	–	–	–
**towns/villages**	0.91 (0.7-1.18)	–	–	–	–
**Occupation**					
**Non-agricultural**	Ref		Ref	Ref	Ref
**Agricultural**	0.65 (0.45-0.94)*		–	–	–
**Smoking status**					
**No**	Ref		Ref	Ref	Ref
**Yes**	1.16 (0.99-1.37)		–	–	–
**Alcohol consumption**					
**No**	Ref		Ref	Ref	Ref
**Yes**	0.74 (0.64-0.86)***		0.71 (0.61-0.83)***	0.76 (0.64-0.89)***	0.77 (0.65-0.91)**
**Sleep duration**					
**< 6 hours**	5.95 (5.08-6.98)***			5.46 (4.63-6.43)***	5.42 (4.61-6.39)***
**6–8 hours**	Ref			Ref	Ref
**≥ 8 hours**	0.58 (0.39-0.87)**			–	–
**Energy (kcal/day)**	1.00 (1.00-1.00)				–
**Protein (% energy)**	0.98 (0.95-1.00)*				–
**Fat (% energy)**	1.02 (1.01-1.03)***				–
**Vitamin D intake (μg/1000 kcal)**	0.96 (0.94-0.98)***				–
**Magnesium intake (mg/1000 kcal)**	0.99 (0.99-1.00)***				–

Odds ratios (ORs) and 95% confidence intervals (CIs) for NRS according to insoluble dietary fiber intake and covariates. Logistic regression analyses were conducted for the unadjusted model; Model 1 (adjusted for sex, age, and BMI); Model 2 (further adjusted for household size, household income, city size, occupation, smoking status, and alcohol consumption); Model 3 (additionally adjusted for sleep duration); and Model 4 (further adjusted for dietary factors, including total energy intake, protein, fat, vitamin D, and magnesium intake). Statistical significance was defined as *p < 0.05, **p < 0.01, and ***p < 0.001. A dash ("–") indicates variables that were not included in the respective model.

Table S2. Logistic regression analysis of factors associated with NRS using multiple imputation.

Variable	Unadjusted OR (95%CI)	Model 1 AOR (95%CI)	Model 2 AOR (95%CI)	Model 3 AOR (95%CI)	Model 4 AOR (95%CI)
**Insoluble dietary fiber (g/1,000kcal)**	0.90 (0.87-0.92)***	0.94 (0.92-0.96)***	0.94 (0.92-0.97)***	0.94 (0.92-0.97)***	0.97 (0.94-1.00)*
**Sex**					
**Male**	Ref	Ref	Ref	Ref	Ref
**Female**	1.01 (0.93-1.09)	–	1.12 (1.01-1.23)*	–	–
**Age**					
**< 60 years**	Ref	Ref	Ref	Ref	Ref
**≥ 60 years**	0.36 (0.33-0.39)***	0.38 (0.35-0.42)***	0.41 (0.37-0.45)***	0.48 (0.43-0.53)***	0.46 (0.41-0.51)***
**BMI**	0.99 (0.98-1.01)	–	–	–	–
**Household size**					
**1 person**	Ref		Ref	Ref	Ref
**≥ 2 persons**	1.12 (0.99-1.27)		0.79 (0.69-0.91)***	0.84 (0.73-0.98)*	–
**Household income**					
**< 2 million yen**	Ref		Ref	Ref	Ref
**2–6 million yen**	1.25 (1.09-1.45)**		–	–	–
**≥ 6 million yen**	1.87 (1.56-2.24)***		1.56 (1.27-1.91)***	1.44 (1.15-1.81)**	1.41 (1.13-1.77)**
**Occupation**					
**Non-agricultural**	Ref		Ref	Ref	Ref
**Agricultural**	0.58 (0.45-0.75)***		–	–	–
**Smoking status**					
**No**	Ref		Ref	Ref	Ref
**Yes**	1.40 (1.23-1.59)***		1.19 (1.03-1.38)*	–	–
**Alcohol consumption**					
**No**	Ref		Ref	Ref	Ref
**Yes**	0.89 (0.79-1.00)		0.83 (0.73-0.94)**	0.86 (0.74-0.99)*	–
**Sleep duration**					
**< 6 hours**	5.55 (4.93-6.25)***			5.30 (4.71-5.98)***	5.44 (4.83-6.13)***
**6–8 hours**	Ref			Ref	Ref
**≥ 8 hours**	0.52 (0.34-0.80)**			–	–
**Energy (kcal/day)**	1.00 (1.00-1.00)				–
**Protein (% energy)**	0.97 (0.95-0.98)***				–
**Fat (% energy)**	1.02 (1.01-1.02)***				–
**Vitamin D intake (μg/1000 kcal)**	0.97 (0.96-0.98)***				–
**Magnesium intake (mg/1000 kcal)**	0.99 (0.99-1.00)***				–

Odds ratios (ORs) and 95% confidence intervals (CIs) for NRS according to insoluble dietary fiber intake and covariates. Logistic regression analyses were conducted for the unadjusted model; Model 1 (adjusted for sex, age, and BMI); Model 2 (further adjusted for household size, household income, occupation, smoking status, and alcohol consumption); Model 3 (additionally adjusted for sleep duration); and Model 4 (further adjusted for dietary factors, including total energy intake, protein, fat, vitamin D, and magnesium intake). Statistical significance was defined as *$p < 0.05$, **$p < 0.01$, and ***$p < 0.001$. A dash ("–") indicates variables that were not included in the respective model.

Table S3-1. Unadjusted multinomial logistic regression analysis of factors associated with NRS.

Variable	Unadjusted OR (95%CI)		
	1 vs 4	1 vs 3	1 vs 2
**Insoluble dietary fiber (g/1,000kcal)**	0.74 (0.66-0.83)***	0.87 (0.83-0.90)***	0.96 (0.94-0.99)**
**Sex**			
**Male**	Ref	Ref	Ref
**Female**	0.77 (0.45-1.33)	1.18 (0.97-1.43)	1.19 (1.02-1.38)*
**Age**			
**< 60 years**	Ref	Ref	Ref
**≥ 60 years**	0.07 (0.04-0.11)***	0.18 (0.15-0.22)***	0.46 (0.39-0.53)***
**BMI**	0.98 (0.92-1.04)	1.00 (0.98-1.03)	0.99 (0.97-1.01)
**Household size**			
**1 person**	Ref	Ref	Ref
**≥ 2 persons**	2.12 (1.14-3.94)*	1.09 (0.89-1.33)	1.11 (0.95-1.30)
**Household income**			
**< 2 million yen**	Ref	Ref	Ref
**2–6 million yen**	1.65 (0.88-3.12)	1.31 (1.05-1.62)*	1.29 (1.10-1.51)**
**≥ 6 million yen**	3.64 (1.88-7.03)***	2.39 (1.86-3.06)***	1.49 (1.23-1.81)***
**Occupation**			
**Non-agricultural**	Ref	Ref	Ref
**Agricultural**	0.19 (0.03-1.40)	0.66 (0.43-1.00)*	0.91 (0.68-1.21)
**Smoking status**			
**No**	Ref	Ref	Ref
**Yes**	1.61 (1.02-2.56)*	1.17 (0.96-1.43)	1.06 (0.91-1.23)
**Alcohol consumption**			
**No**	Ref	Ref	Ref
**Yes**	0.73 (0.47-1.14)	0.67 (0.57-0.80)***	0.87 (0.76-0.99)*
**Sleep duration**			
**< 6 hours**	55.13 (26.17-116.16)***	12.86 (10.30-16.05)***	3.14 (2.61-3.77)***
**6–8 hours**	Ref	Ref	Ref
**≥ 8 hours**	—	0.34 (0.23-0.51)***	0.33 (0.27-0.41)***
**Energy (kcal/day)**	1.00 (1.00-1.00)	1.00 (1.00-1.00)	1.00 (1.00-1.00)
**Protein (% energy)**	0.97 (0.90-1.04)	0.96 (0.94-0.99)**	0.98 (0.96-1.00)
**Fat (% energy)**	1.02 (1.00-1.05)	1.03 (1.02-1.05)***	1.02 (1.01-1.03)***
**Vitamin D intake (μg/1000 kcal)**	0.92 (0.86-0.98)*	0.95 (0.93-0.97)***	0.98 (0.97-1.00)*
**Magnesium intake (mg/1000 kcal)**	0.99 (0.98-0.99)***	1.00 (0.99-1.00)***	1.00 (1.00-1.00)

Table S3-2. Adjusted multinomial logistic regression analysis of factors associated with NRS (Model 1).

Variable	Model 1 AOR (95%CI)		
	1 vs 4	1 vs 3	1 vs 2
**Insoluble dietary fiber (g/1,000kcal)**	0.87 (0.77-0.99)*	0.95 (0.91-0.99)*	–
**Sex**			
**Male**	Ref	Ref	Ref
**Female**	–	1.45 (1.18-1.80)***	1.26 (1.08-1.48)**
**Age**			
**< 60 years**	Ref	Ref	Ref
**≥ 60 years**	0.08 (0.04-0.14)***	0.19 (0.16-0.23)***	0.46 (0.39-0.53)***
**BMI**	–	—	—

Table S3-3. Adjusted multinomial logistic regression analysis of factors associated with NRS (Model 2).

Variable	Unadjusted OR (95%CI)		
	1 vs 4	1 vs 3	1 vs 2
**Insoluble dietary fiber (g/1,000kcal)**	0.86 (0.76-0.97)*	0.93 (0.89-0.97)**	–
**Sex**			
**Male**	Ref	Ref	Ref
**Female**	–	1.28 (1.00-1.63)*	1.31 (1.08-1.58)**
**Age**			
**< 60 years**	Ref	Ref	Ref
**≥ 60 years**	0.08 (0.05-0.15)***	0.20 (0.16-0.25)***	0.47 (0.39-0.55)***
**BMI**	–	–	–
**Household size**			
**1 person**	Ref	Ref	Ref
**≥ 2 persons**	–	–	–
**Household income**			
**< 2 million yen**	Ref	Ref	Ref
**2–6 million yen**	–	–	1.24 (1.04-1.48)*
**≥ 6 million yen**	–	–	–
**Occupation**			
**Non-agricultural**	Ref	Ref	Ref
**Agricultural**	–	–	–
**Smoking status**			
**No**	Ref	Ref	Ref
**Yes**	–	–	–
**Alcohol consumption**			
**No**	Ref	Ref	Ref
**Yes**	0.63 (0.39-1.00)*	0.66 (0.55-0.80)***	–

Table S3-4. Adjusted multinomial logistic regression analysis of factors associated with NRS (Model 3).

Variable	Unadjusted OR (95%CI)		
	1 vs 4	1 vs 3	1 vs 2
**Insoluble dietary fiber (g/1,000kcal)**	0.87 (0.76-0.98)*	0.93 (0.89-0.98)**	–
**Sex**			
**Male**	Ref	Ref	Ref
**Female**	–	–	–
**Age**			
**< 60 years**	Ref	Ref	Ref
**≥ 60 years**	0.11 (0.06-0.21)***	0.25 (0.20-0.32)***	0.54 (0.45-0.64)***
**BMI**	–	–	–
**Household size**			
**1 person**	Ref	Ref	Ref
**≥ 2 persons**	–	–	–
**Household income**			
**< 2 million yen**	Ref	Ref	Ref
**2–6 million yen**	–	–	–
**≥ 6 million yen**	–	–	–
**Occupation**			
**Non-agricultural**	Ref	Ref	Ref
**Agricultural**	–	–	–
**Smoking status**			
**No**	Ref	Ref	Ref
**Yes**	–	–	–
**Alcohol consumption**			
**No**	Ref	Ref	Ref
**Yes**	–	0.69 (0.56-0.85)***	–
**Sleep duration**			
**< 6 hours**	51.46 (24.29-109.04)***	11.99 (9.55-15.06)***	3.10 (2.57-3.74)***
**6–8 hours**	Ref	Ref	Ref
**≥ 8 hours**	–	0.44 (0.29-0.68)***	0.37 (0.30-0.45)***

Table S3-5. Adjusted multinomial logistic regression analysis of factors associated with NRS (Model 4).

Variable	Unadjusted OR (95%CI)		
	1 vs 4	1 vs 3	1 vs 2
**Insoluble dietary fiber (g/1,000kcal)**	–	0.93 (0.88-0.99)*	–
**Sex**			
**Male**	Ref	Ref	Ref
**Female**	–	–	–
**Age**			
**< 60 years**	Ref	Ref	Ref
**≥ 60 years**	0.12 (0.06-0.22)***	0.26 (0.21-0.33)***	0.55 (0.46-0.65)***
**BMI**	–	–	–
**Household size**			
**1 person**	Ref	Ref	Ref
**≥ 2 persons**	–	–	–
**Household income**			
**< 2 million yen**	Ref	Ref	Ref
**2–6 million yen**	–	–	–
**≥ 6 million yen**	–	–	–
**Occupation**			
**Non-agricultural**	Ref	Ref	Ref
**Agricultural**	–	–	–
**Smoking status**			
**No**	Ref	Ref	Ref
**Yes**	–	–	–
**Alcohol consumption**			
**No**	Ref	Ref	Ref
**Yes**	–	0.70 (0.57-0.86)***	–
**Sleep duration**			
**< 6 hours**	50.33 (23.74-106.72)***	11.94 (9.50-15.00)***	3.10 (2.57-3.74)***
**6–8 hours**	Ref	Ref	Ref
**≥ 8 hours**	–	0.45 (0.29-0.68)***	0.37 (0.30-0.46)***
**Energy (kcal/day)**	–	–	0.97 (0.94-1.00)*
**Protein (% energy)**	–	–	1.01 (1.00-1.02)*
**Fat (% energy)**	–	–	–
**Vitamin D intake (μg/1000 kcal)**	–	–	–
**Magnesium intake (mg/1000 kcal)**	–	–	–

Odds ratios (ORs) and 95% confidence intervals (CIs) for the four-category NRS outcome were estimated using multinomial logistic regression. NRS was categorized into four levels: 4 = non-restorative sleep (NRS), 3 = low restorative sleep, 2 = fair restorative sleep, and 1 = sufficient restorative sleep (reference category). ORs represent the relative odds of belonging to each NRS level (4, 3, or 2) compared with the reference category (1). Statistical significance was defined as *p < 0.05, **p < 0.01, and *p < 0.001. A dash ("–") indicates that the variable was not included in the respective model or that the estimate could not be computed due to zero cell counts (complete separation).

Table S4. Logistic regression analysis of factors associated with NRS using energy-adjusted insoluble dietary fiber (residual method)

Variable	Unadjusted OR (95%CI)	Model 1 AOR (95%CI)	Model 2 AOR (95%CI)	Model 3 AOR (95%CI)	Model 4 AOR (95%CI)
**Insoluble dietary fiber (g)**	0.93 (0.92-0.95)***	0.97 (0.95-0.99)**	0.97 (0.95-0.98)***	0.97 (0.95-0.99)**	0.97 (0.95-1.00)*
**Sex**					
**Male**	Ref	Ref	Ref	Ref	Ref
**Female**	1.01 (0.93-1.09)	1.19 (1.00-1.41)*	–	–	–
**Age**					
**< 60 years**	Ref	Ref	Ref	Ref	Ref
**≥ 60 years**	0.36 (0.33-0.39)***	0.31 (0.27-0.37)***	0.32 (0.27-0.38)***	0.39 (0.32-0.46)***	0.39 (0.33-0.47)***
**BMI**	0.99 (0.98-1.01)	–	–	–	–
**Household size**					
**1 person**	Ref		Ref	Ref	Ref
**≥ 2 persons**	1.12 (0.99-1.27)		–	–	–
**Household income**					
**< 2 million yen**	Ref		Ref	Ref	Ref
**2–6 million yen**	1.25 (1.09-1.45)**		–	–	–
**≥ 6 million yen**	1.87 (1.56-2.24)***		–	–	–
**Occupation**					
**Non-agricultural**	Ref		Ref	Ref	Ref
**Agricultural**	0.58 (0.45-0.75)***		–	–	–
**Smoking status**					
**No**	Ref		Ref	Ref	Ref
**Yes**	1.40 (1.23-1.59)***		–	–	–
**Alcohol consumption**					
**No**	Ref		Ref	Ref	Ref
**Yes**	0.89 (0.79-1.00)		0.71 (0.61-0.83)***	0.75 (0.64-0.89)***	0.77 (0.65-0.91)**
**Sleep duration**					
**< 6 hours**	5.55 (4.93-6.25)***			5.44 (4.62-6.40)***	5.41 (4.60-6.37)***
**6–8 hours**	Ref			Ref	Ref
**≥ 8 hours**	0.52 (0.34-0.80)**			–	–
**Energy (kcal/day)**	1.00 (1.00-1.00)				–
**Protein (g)**	1.00 (0.99-1.00)*				–
**Fat (g)**	1.00 (1.00-1.00)*				–
**Vitamin D intake (μg)**	0.98 (0.97-0.99)***				–
**Magnesium intake (mg)**	1.00 (1.00-1.00)***				–

Odds ratios (ORs) and 95% confidence intervals (CIs) for NRS according to insoluble dietary fiber intake and covariates. Insoluble dietary fiber, protein, fat, vitamin D, and magnesium intakes were energy-adjusted using the residual method. Logistic regression analyses were conducted for the unadjusted model; Model 1 (adjusted for sex, age, and BMI); Model 2 (further adjusted for household size, household income, occupation, smoking status, and alcohol consumption); Model 3 (additionally adjusted for sleep duration); and Model 4 (further adjusted for dietary factors, including total energy intake, protein, fat, vitamin D, and magnesium intake). Statistical significance was defined as *$p < 0.05$, **$p < 0.01$, and ***$p < 0.001$. A dash ("–") indicates variables that were not included in the respective model.

Table S5. Logistic regression analysis of factors associated with NRS, incorporating age as a continuous variable and survey year (2014–2018) as an additional covariate.

Variable	Unadjusted OR (95%CI)	Model 1 AOR (95%CI)	Model 2 AOR (95%CI)	Model 3 AOR (95%CI)	Model 4 AOR (95%CI)
**Insoluble dietary fiber (g/1,000kcal)**	0.88 (0.85–0.91)***	0.95 (0.92-0.98)**	0.94 (0.91-0.98)**	0.95 (0.91-0.99)**	0.95 (0.91-0.99)*
**Sex**					
**Male**	Ref	Ref	Ref	Ref	Ref
**Female**	1.01 (0.86-1.19)	1.20 (1.01-1.42)*	–	–	–
**Age**	0.96 (0.96-0.97)***	0.96 (0.96-0.97)***	0.97 (0.96-0.97)***	0.97 (0.96-0.98)***	0.97 (0.96-0.98)***
**BMI**	1.01 (0.99-1.03)	–	–	–	–
**Survey year**					
**2018**	Ref				
**2014**	0.87 (0.76-1.00)				
**Household size**					
**1 person**	Ref		Ref	Ref	Ref
**≥ 2 persons**	1.07 (0.90-1.26)		–	–	–
**Household income**					
**< 2 million yen**	Ref		Ref	Ref	Ref
**2–6 million yen**	1.13 (0.94-1.36)		–	–	–
**≥ 6 million yen**	1.90 (1.55-2.33)***		1.30 (1.02-1.66)*	–	–
**Occupation**					
**Non-agricultural**	Ref		Ref	Ref	Ref
**Agricultural**	0.65 (0.45-0.94)*		–	–	–
**Smoking status**					
**No**	Ref		Ref	Ref	Ref
**Yes**	1.16 (0.99-1.37)		–	–	–
**Alcohol consumption**					
**No**	Ref		Ref	Ref	Ref
**Yes**	0.74 (0.64-0.86)***		0.75 (0.64-0.87)***	0.79 (0.67-0.93)**	0.80 (0.67-0.94)**
**Sleep duration**					
**< 6 hours**	5.95 (5.08-6.98)***			5.53 (4.70-6.51)***	5.50 (4.67-6.48)***
**6–8 hours**	Ref			Ref	Ref
**≥ 8 hours**	0.58 (0.39-0.87)**			–	–
**Energy (kcal/day)**	1.00 (1.00-1.00)				–
**Protein (% energy)**	0.98 (0.95-1.00)*				–
**Fat (% energy)**	1.02 (1.01-1.03)***				–
**Vitamin D intake (μg/1000 kcal)**	0.96 (0.94-0.98)***				–
**Magnesium intake (mg/1000 kcal)**	0.99 (0.99-1.00)***				–

Odds ratios (ORs) and 95% confidence intervals (CIs) for NRS according to insoluble dietary fiber intake and covariates. Logistic regression analyses were conducted for the unadjusted model; Model 1 (adjusted for sex, age, and BMI); Model 2 (further adjusted for Survey year, household size, household income, occupation, smoking status, and alcohol consumption); Model 3 (additionally adjusted for sleep duration); and Model 4 (further adjusted for dietary factors, including total energy intake, protein, fat, vitamin D, and magnesium intake). Statistical significance was defined as *$p < 0.05$, **$p < 0.01$, and ***$p < 0.001$. A dash (“–”) indicates variables that were not included in the respective model.

Table S6. Logistic regression analysis of factors associated with NRS using detailed occupation categories instead of dichotomized occupation status.

Variable	Unadjusted OR (95%CI)	Model 1 AOR (95%CI)	Model 2 AOR (95%CI)	Model 3 AOR (95%CI)	Model 4 AOR (95%CI)
**Insoluble dietary fiber (g/1,000kcal)**	0.88 (0.85–0.91)***	0.95 (0.91-0.98)**	0.94 (0.90-0.97)***	0.95 (0.91-0.98)**	0.95 (0.91-1.00)*
**Sex**					
**Male**	Ref	Ref	Ref	Ref	Ref
**Female**	1.01 (0.86-1.19)	1.20 (1.01-1.43)*	–	–	–
**Age**					
**< 60 years**	Ref	Ref	Ref	Ref	Ref
**≥ 60 years**	0.29 (0.25-0.34)***	0.31 (0.27-0.36)***	0.36 (0.29-0.43)***	0.41 (0.33-0.49)***	0.41 (0.34-0.51)***
**BMI**	1.01 (0.99-1.03)	–	–	–	–
**Household size**					
**1 person**	Ref		Ref	Ref	Ref
**≥ 2 persons**	1.07 (0.90-1.26)		–	–	–
**Household income**					
**< 2 million yen**	Ref		Ref	Ref	Ref
**2–6 million yen**	1.13 (0.94-1.36)		–	–	–
**≥ 6 million yen**	1.90 (1.55-2.33)***		–	–	–
**Employment status**					
**Unemployed**	Ref		Ref	Ref	Ref
**Employed**	2.29 (1.95-2.69)***		1.27 (1.03-1.56)*	–	–
**Smoking status**					
**No**	Ref		Ref	Ref	Ref
**Yes**	1.16 (0.99-1.37)		–	–	–
**Alcohol consumption**					
**No**	Ref		Ref	Ref	Ref
**Yes**	0.74 (0.64-0.86)***		0.71 (0.60-0.83)***	0.75 (0.64-0.89)***	0.76 (0.64-0.90)**
**Sleep duration**					
**< 6 hours**	5.95 (5.08-6.98)***			5.42 (4.61-6.38)***	5.39 (4.58-6.34)***
**6–8 hours**	Ref			Ref	Ref
**≥ 8 hours**	0.58 (0.39-0.87)**			–	–
**Energy (kcal/day)**	1.00 (1.00-1.00)				–
**Protein (% energy)**	0.98 (0.95-1.00)*				–
**Fat (% energy)**	1.02 (1.01-1.03)***				–
**Vitamin D intake (μg/1000 kcal)**	0.96 (0.94-0.98)***				–
**Magnesium intake (mg/1000 kcal)**	0.99 (0.99-1.00)***				–

Odds ratios (ORs) and 95% confidence intervals (CIs) for NRS according to insoluble dietary fiber intake and covariates. Logistic regression analyses were conducted for the unadjusted model; Model 1 (adjusted for sex, age, and BMI); Model 2 (further adjusted for household size, household income, employment status , smoking status, and alcohol consumption); Model 3 (additionally adjusted for sleep duration); and Model 4 (further adjusted for dietary factors, including total energy intake, protein, fat, vitamin D, and magnesium intake). Statistical significance was defined as *p < 0.05, **p < 0.01, and ***p < 0.001. A dash (“–”) indicates variables that were not included in the respective model.

Table S7. Logistic regression analysis of factors associated with NRS using quartiles of insoluble dietary fiber intake.

Variable	Unadjusted OR (95%CI)	Model 1 AOR (95%CI)	Model 2 AOR (95%CI)	Model 3 AOR (95%CI)	Model 4 AOR (95%CI)
**Insoluble dietary fiber (g/1,000kcal)**					
**Q1**	Ref	Ref	Ref	Ref	Ref
**Q2**	0.75 (0.62-0.91)**	–	–	–	–
**Q3**	0.59 (0.49-0.72)***	0.80 (0.65-0.98)*	0.76 (0.62-0.94)*	–	–
**Q4**	0.48 (0.39-0.59)***	0.74 (0.60-0.92)**	0.70 (0.56-0.87)**	0.73 (0.58-0.93)*	–
**Sex**					
**Male**	Ref	Ref	Ref	Ref	Ref
**Female**	1.01 (0.86-1.19)	1.19 (1.01-1.42)*	–	–	–
**Age**					
**< 60 years**	Ref	Ref	Ref	Ref	Ref
**≥ 60 years**	0.29 (0.25-0.34)***	0.31 (0.27-0.36)***	0.32 (0.27-0.38)***	0.38 (0.32-0.46)***	0.39 (0.33-0.47)***
**BMI**	1.01 (0.99-1.03)	–	–	–	–
**Household size**					
**1 person**	Ref		Ref	Ref	Ref
**≥ 2 persons**	1.07 (0.90-1.26)		–	–	–
**Household income**					
**< 2 million yen**	Ref		Ref	Ref	Ref
**2–6 million yen**	1.13 (0.94-1.36)		–	–	–
**≥ 6 million yen**	1.90 (1.55-2.33)***		–	–	–
**Occupation**					
**Non-agricultural**	Ref		Ref	Ref	Ref
**Agricultural**	0.65 (0.45-0.94)*		–	–	–
**Smoking status**					
**No**	Ref		Ref	Ref	Ref
**Yes**	1.16 (0.99-1.37)		–	–	–
**Alcohol consumption**					
**No**	Ref		Ref	Ref	Ref
**Yes**	0.74 (0.64-0.86)***		0.71 (0.61-0.83)***	0.76 (0.64-0.89)**	0.77 (0.65-0.91)**
**Sleep duration**					
**< 6 hours**	5.95 (5.08-6.98)***			5.43 (4.62-6.40)***	5.40 (4.59-6.36)***
**6–8 hours**	Ref			Ref	Ref
**≥ 8 hours**	0.58 (0.39-0.87)**			–	–
**Energy (kcal/day)**	1.00 (1.00-1.00)				–
**Protein (% energy)**	0.98 (0.95-1.00)*				–
**Fat (% energy)**	1.02 (1.01-1.03)***				–
**Vitamin D intake (µg/1000 kcal)**	0.96 (0.94-0.98)***				–
**Magnesium intake (mg/1000 kcal)**	0.99 (0.99-1.00)***				–

Odds ratios (ORs) and 95% confidence intervals (CIs) for NRS (defined as the absence of sufficient restorative sleep) according to quartiles of insoluble dietary fiber intake. Insoluble dietary fiber intake was categorized into quartiles (Q1–Q4), with Q1 representing the lowest intake group and Q4 the highest intake group. Logistic regression models included the unadjusted model; Model 1 (adjusted for sex, age, and BMI); Model 2 (further adjusted for household size, household income, occupation, smoking status, and alcohol consumption); Model 3 (additionally adjusted for sleep duration); and Model 4 (further adjusted for dietary factors, including total energy intake, protein, fat, vitamin D, and magnesium intake). Statistical significance was defined as *p < 0.05, **p < 0.01, and **p < 0.001. A dash ("–") indicates variables that were not included in the respective model.
